# Investigating the effects of excitatory and inhibitory somatosensory rTMS on somatosensory functioning in the acute and subacute phases of stroke: a preliminary double-blind and randomized trial

**DOI:** 10.3389/fnhum.2024.1474212

**Published:** 2024-09-30

**Authors:** Jingtian Gao, Helin Wang, Zhouyao Hu, Jiqing He, Jing Yang, Xiaokun Lou, Zhiyuan You, Jie Li, Jinghua Wang, Zhongming Gao

**Affiliations:** ^1^Centre for Cognition and Brain Disorders, The Affiliated Hospital of Hangzhou Normal University, Hangzhou, China; ^2^School of Clinical Medicine, Hangzhou Normal University, Hangzhou, China; ^3^Department of Obstetrics, The Affiliated Hospital of Hangzhou Normal University, Hangzhou, China; ^4^School of Clinical Medicine, The Affiliated Hospital of Hangzhou Normal University, Hangzhou, China; ^5^Department of Radiology, The Affiliated Hospital of Hangzhou Normal University, Hangzhou, China; ^6^Department of Neurology, The Affiliated Hospital of Hangzhou Normal University, Hangzhou, China

**Keywords:** TMS, motor cortex, stroke, sensation, excitability

## Abstract

**Background:**

Repetitive transcranial magnetic stimulation (rTMS) targeting the primary somatosensory cortex (S1) has a potential effect on somatosensory functioning following a stroke. However, S1-rTMS was combined with peripheral therapies in previous trials. Moreover, these studies have commonly targeted the ipsilesional S1 with excitatory rTMS paradigms.

**Methods:**

This double-blind, randomized trial (registration number: ChiCTR2200059098) investigated two forms of paradigms, that is ipsilesional excitatory and contralesional inhibitory rTMS, as a stand-alone treatment in post-stroke somatosensation. Patients in the acute and subacute phases of stroke were randomly assigned to either contralesional 1-Hz or ipsilesional 10-Hz rTMS group and received 10 daily sessions of treatment in two consecutive weeks.

**Results:**

Results indicate that the contralesional inhibitory and ipsilesional excitatory stimulation were equally effective in improving somatosensory functioning. Moreover, this effect was most prominent in deep sensations and subjective sensations. Using single-pulse EMG recordings, our data also revealed an increased MEP amplitude in the ipsilesional motor cortex following ipsilesional excitatory treatment.

**Conclusion:**

This preliminary study demonstrates the primary somatosensory cortex as an effective rTMS target in somatosensory recovery following stroke.

**Clinical trial registration:**

https://www.chictr.org.cn/showproj.html?proj=166474, ChiCTR2200059098.

## 1 Introduction

Somatosensory deficits are common post-stroke symptoms characterized by sensory loss and numbness. It is estimated that 50–80% of post-stroke survivors experience somatosensory deficits, which have a clear adverse influence on the quality of life (Bolognini et al., 2016; Krause et al., 2016). Repetitive transcranial magnetic stimulation (rTMS) is a safe and non-invasive form of brain stimulation that is able to induce neuroplastic change (Pascual-Leone et al., 1998; Che et al., 2019). rTMS has been used to manage depression (Blumberger et al., 2018; Fitzgerald et al., 2022; Zhao et al., 2024), chronic pain (Attal et al., 2021; Wang et al., 2023; Zhou et al., 2024), and post-stroke rehabilitation (Hosomi et al., 2016; Guan et al., 2017). In post-stroke rehabilitation, studies have predominantly focussed on motor rehabilitation (Lefaucheur et al., 2020). However, the potential benefits of rTMS on somatosensory deficits remain unclear. Moreover, there is a lack of effective treatment for somatosensory deficits following stroke (Schabrun and Hillier, 2009; Carlsson et al., 2018).

A few pilot studies have investigated the effects of rTMS on somatosensory deficits following a stroke. These studies targeted the ipsilesional primary somatosensory cortex (S1) and reported promising effects on somatosensory functioning (Brodie et al., 2014; de Freitas Zanona et al., 2022). However, in one way, S1-rTMS was combined with peripheral therapies in these trials such as sensory stimulation or sensory feedback, although a sham peripheral intervention was carefully designed. In another way, these studies have commonly targeted the ipsilesional S1 with excitatory rTMS paradigms (e.g., 5 or 10 Hz). Nonetheless, these novel clinical trials have indicated the potential of TMS treatment in managing somatosensory deficits following a stroke.

Building on these trials, the current study sought to investigate the effects of S1-rTMS on somatosensory functioning. In one way, S1-rTMS was delivered without a peripheral treatment, which is helpful to clarify the effects of S1-rTMS. In another way, contralesional inhibitory rTMS was also piloted, with the intention of testing an alternative treatment protocol and comparing the efficacy with ipsilesional treatment. It is noted that contralesional inhibitory rTMS was found to enhance motor learning post-stroke (Meehan et al., 2011). In the example of motor recovery, inhibitory rTMS over the contralesional primary motor cortex (M1) has a definite effect on hand motor recovery in the post-acute stage of stroke (Lefaucheur et al., 2020). A line of evidence suggested that poststroke hyperexcitability of the contralesional hemisphere may decrease the excitability of the ipsilesional hemisphere, thus representing a poor prognostic factor for clinical outcomes (Traversa et al., 1998).

This preliminary, double-blind, and randomized piloting trial was designed to investigate the effects of S1-rTMS on post-stroke somatosensory deficits. In order to understand the corticospinal mechanisms, corticospinal excitation and inhibition were measured with single-pulse electromyography (EMG) protocol of motor-evoked potential (MEP) and cortical silent period (CSP) respectively. MEP amplitude provides a simple and direct measurement of the excitation of corticospinal pathways. Meanwhile, CSP is able to evaluate intracortical inhibition supported by gamma-aminobutyric acid (GABA_B_) mediated neurotransmission (Werhahn et al., 1999). According to the literature, it is hypothesized that both ipsilesional excitatory and contralesional inhibitory rTMS would be effective to manage post-stroke somatosensory deficits.

## 2 Materials and methods

### 2.1 Participants

Potential participants were recruited from the Affiliated Hospital of Hangzhou Normal University from April 2022 to August 2022. All patients had unilateral ischemic stroke observed on a diffusion-weighted MRI scan. The inclusion criteria were: (1) unilateral ischemic stroke in the acute and subacute phase of stroke (< 6 months) (Lefaucheur et al., 2014); and (2) with somatosensory deficits caused by ischemic stroke; and (3) 18–80 years old; and (4) were on regular stroke medicines. The exclusion criteria were: (1) TMS contradictions such as current or a history of seizure or implanted devices (pacemaker, medical pumps) (Rossi et al., 2011); or (2) severe mental disorders assessed with HAMD or HAMA (Hamilton, 1959, 1967); or (3) aphasia or cognitive disorders assessed with MMSE (Folstein et al., 1975); or (4) not able to communicate with a doctor; or (5) severe disorders caused by other conditions such as tumour or severe heart or lung malfunctioning; or (6) mRS > 2; or (7) somatosensory deficits not caused by ischemic stroke, e.g., diabetes pain. mRS was used to include mostly mild impairment as somatosensory deficits are more prominent for these patients compared to motor deficits in patients with severe stroke. The withdrawal criteria were: (1) changes in medication after allocation; or (2) newly diagnosed stroke or other neurological lesions after allocation; or (3) that patients decided to withdraw from the study.

### 2.2 Study overview

We conducted an open label and randomized trial registered in the Chinese Clinical Trials Registry (ChiCTR2200059098). Patients were randomly assigned to either contralesional 1-Hz or ipsilesional 10-Hz rTMS treatment according to a centrally stratified computer-generated randomization protocol by ZG. Allocation concealment was performed independently by a staff member (JG). Patients received 10 daily sessions in two consecutive weeks delivered by a trained neurologist (ZH). Clinical assessments were performed at pre-and post-treatment by a blinded neurologist (ZG). All participants provided written informed consent before the study commencement. This study was approved by the Ethics Committee in the Affiliated Hospital of Hangzhou Normal University (2021-E2-HS-029) and was conducted in accordance with the Declaration of Helsinki.

### 2.3 Somatosensory functioning

Somatosensory functioning was evaluated using the modified Fugl-Meyer and Lindmark Assessment (Fugl-Meyer et al., 1975; Lindmark and Hamrin, 1988), which included superficial sensation (pain, temperature, and touch), deep sensation (proprioception, motion perception, and vibration), cortical sensation (two-point discrimination, stereognosis), and subjective sensation. The subjective sensation was added due to the fact that patients tended to report subjective somatosensory deficits that could not be characterized by the three sensory dimensions (superficial, deep, or cortical sensation) (Gao et al., 2023). The assessment of somatosensory function systematically covered the trunk, limbs, and head with 29 items (superficial = 10; deep = 13, cortical = 3, and subjective = 3), and had a total score of 58 (three-step scale: 0, 1, 2). The score of somatosensory functioning assessed both the ipsilesional and contralesional sides, whereby deficit scores (determined by deficit item and level) were deducted from the total score of 58 (Chen et al., 2019). It is noted that motor functioning was not systematically evaluated with Fugl-Meyer Assessment (FMA) as these patients generally had no complaints about motor functioning compared to somatosensory deficits during the visit to our hospital.

### 2.4 Resting motor threshold and corticospinal excitability

The resting motor threshold (RMT) over the ipsilesional motor cortex was assessed. RMT was defined as the minimum intensity to induce motor-evoked potentials (MEPs) > 0.05 mV of the first dorsal interosseous (FDI) muscle in 5/10 trials. Single pulses to the hand region of the motor cortex (45° to the midline, handle pointing backward) at 5 s ± 10% jitter intervals were sent by a figure-eight coil connected to a Magstim Rapid2 system (Magstim Company Ltd., UK).

Corticospinal excitability was measured with MEP amplitude and CSP latency at rest and during a sustained voluntary FDI muscle contraction, respectively, (Hupfeld et al., 2020; Liu et al., 2022). A total of 40 single pulses (20 of MEP, 20 of CSP) were consecutively delivered to the hand region of the motor cortex at 110% RMT (45° to the midline, handle pointing backward). It is worth noting that CSP was evaluated following MEP as the muscle contraction during CSP may have an impact on resting MEP amplitude (Conforto et al., 2004). Corticospinal excitability was evaluated from the ipsilesional motor cortex.

### 2.5 rTMS treatment

Patients received ten daily sessions of either contralesional 1-Hz or ipsilesional 10-Hz rTMS treatment in two weeks. Each rTMS session delivered 1,500 pulses over the S1 at 80% RMT (Conforto et al., 2012). The 10-Hz protocol delivered 5-s trains with 25-s intervals, while consecutive 1-Hz pulses were delivered in the contralesional 1-Hz group. The S1 was located 2 cm lateral and 0.5 cm posterior to the M1-FDI scalp location. This localisation method was proposed by a recent study across a series of experimental investigations (Holmes et al., 2019). Coil position was measured relative to the nasion and inion to facilitate consistent re-positioning of the coil between sessions (Chung et al., 2019; Ye et al., 2022; Wang et al., 2023).

### 2.6 Data analysis

The total score of somatosensory functioning was analysed as the primary outcome measure, while the subdimensions of somatosensory functioning and corticospinal excitability were set as the secondary outcome measures. Intent-to-treat (ITT) analysis was set to include all randomized patients. This method preserves the benefits of randomization and allows to draw unbiased conclusions regarding the effectiveness of an intervention. Multiple imputation algorithm was initially performed, which is a highly recommended methodology for dealing with missing data (McCoy, 2017). Specifically, we initially created 10 sets of imputations for the missing values. We then perform the desired analysis (i.e., ANOVA) separately for each dataset. We then combined the results from the separate analyses to obtain a single set of estimates.

MEP amplitude was calculated by peak-to-peak amplitude. The calculation of CSP latency was based on the Mean Consecutive Difference (MCD) (Garvey et al., 2001; Wang et al., 2024), which was highly recommended by a recent expert review (Hupfeld et al., 2020). The MCD methodology is briefly described here: (1) All silent period trials were rectified using the absolute value and then were averaged; (2) The MCD of 100 ms EMG data before a TMS pulse was calculated, in which the MCD is the mean successive difference between individual data points; (3) Thresholds were set at: ± MCD × 2.66 (i.e., 3 standard deviations), which covers 99.76% of possible pre-TMS EMG data points; (4) Silent period onset was determined as the time point at which the post-TMS EMG falls below the variation threshold for three consecutive data points, while the silent period offset was defined as the point at which the post-TMS EMG returns above the variation threshold for three consecutive data points.

### 2.7 Statistical analysis

Data were analysed in SPSS (v.25.0 Chicago, Illinois, United States). Demographic variables were initially examined with a two-sample *t*-test or *χ*^2^ test. A series of tests were performed to check the assumptions of using a mixed-design ANOVA. Specifically, the Shapiro–Wilk test was performed to check the normality of the outcome measures in different combinations of our two factors. Levene’s test for homogeneity of variances and Mauchly’s Test of Sphericity were also performed. Results validated the use of mixed ANOVA.

Two-way mixed-design ANOVAs were then performed to examine the main and interaction effects of Group (2 levels: contralesional 1-Hz, ipsilesional 10-Hz) and Time (2 levels: pre, post). *Post-hoc* pairwise comparisons were conducted to further explore the significant main and interaction effects, with the *α*-level set to 0.05 and Bonferroni corrected.

## 3 Results

### 3.1 Clinical characteristics

**Figure 1:**
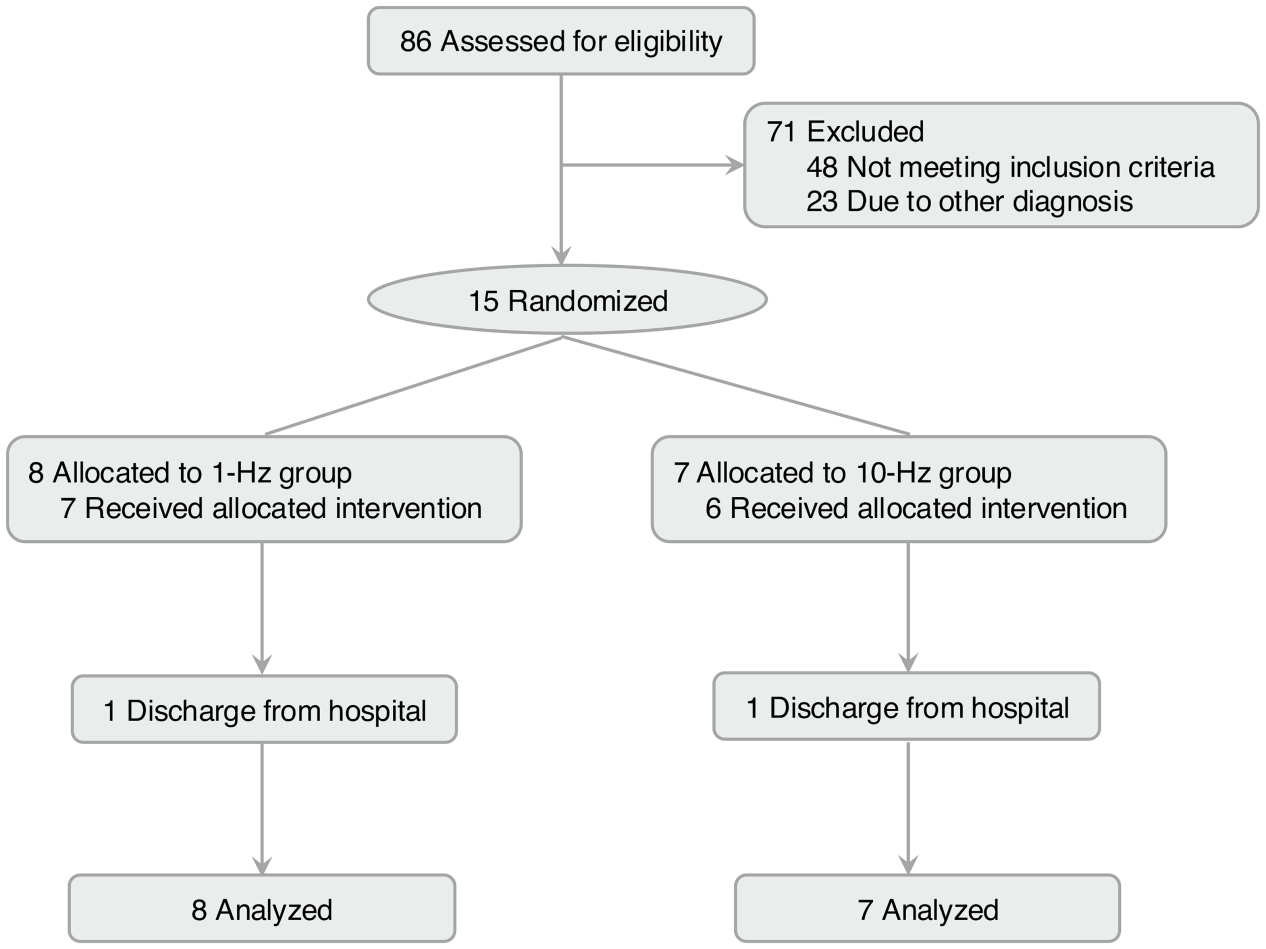
Flow diagram of this study.

A total of 86 patients were screened, of which 71 were excluded due to noting meeting the inclusion criteria (*n* = 48) or not willing to participate (*n* = 23, Figure 1). Fifteen participants were enrolled and equally randomized to the two groups. One patient withdrew from each group due to discharge from the hospital (both in week 2). Data from 15 patients in each group were analysed with ITT methodology.

The demographics and clinical information of patients were presented in Table 1. Patients were mainly old adults with a mean age of 62. Lesions of the brain were mainly distributed in the thalamus, followed by damage to the brainstem and cortical regions such as the temporal and frontoparietal cortex. Somatosensory functioning had a mean score of 47.53 out of a total score of 58. Patients had normal (5-degree, *n* = 11/15) or slightly impaired (4-degree, *n* = 4/15) muscle strength as indexed by the muscle strength grading scale (0–5). The two groups were comparable in all these variables. RMT was also comparable between the two groups (*t* = −0.42, *p* = 0.679). Regular stroke medicines included a daily dose of Aspirin (100 mg) and Atorvastatin (20 mg) for each patient.

**Table 1:** Demographic information of participants.

Measure	1-Hz (*n* = 8)	10-Hz (*n* = 7)	*t*/*χ*^2^	*P*
Age, *y*				
Mean ± SD	62.38 ± 11.33	62.29 ± 10.45	0.02	0.99
Sex				
Male	6	4	0.54	0.46
Female	2	3		
Time from stroke, *m*				
Mean ± SD	1.88 ± 1.64	2.21 ± 1.68	−0.39	0.70
Somatosensory function				
Mean ± SD	47.75 ± 5.39	47.29 ± 10.19	0.11	0.92
Muscle strength				
Mean ± SD	4.75 ± 0.46	4.57 ± 0.53	0.69	0.51
Lesion, *n*				
Thalamus	5	2		
Pons	1	3		
Temporal	1	0		
Frontoparietal	1	1		
Medulla	0	1		

y, year; m, month.

### 3.2 Treatment efficacy

**Figure 2:**
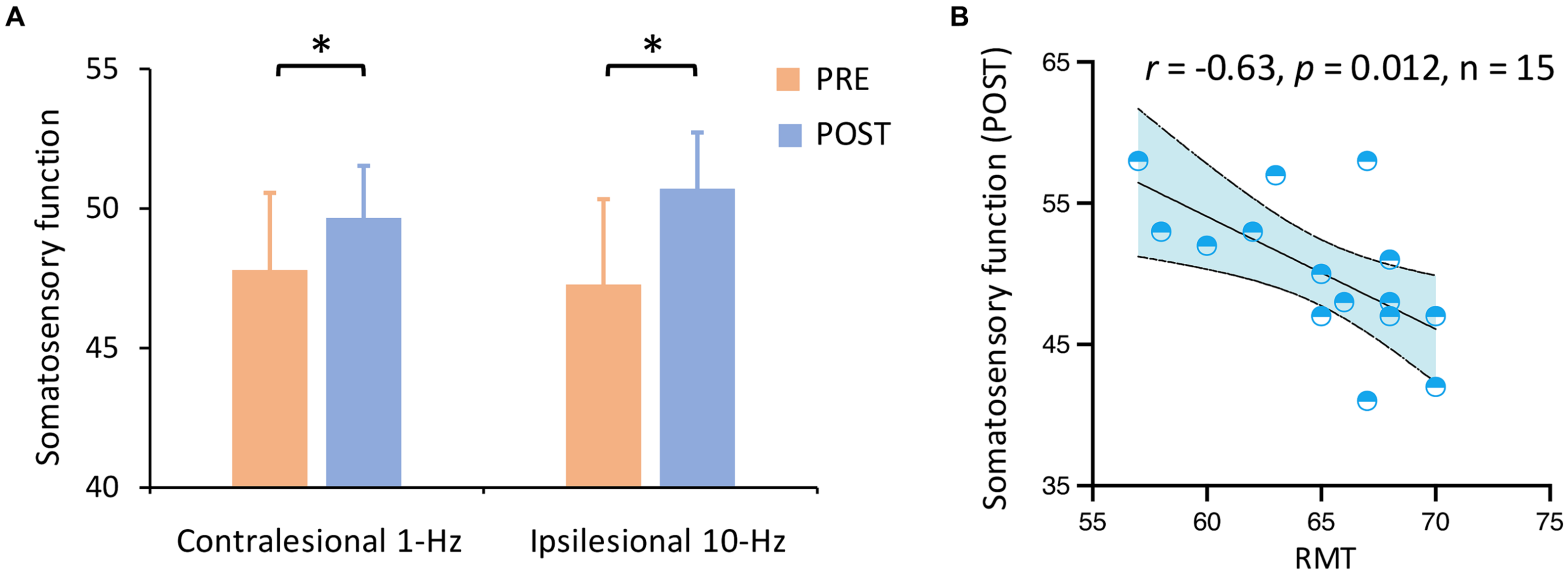
Somatosensory function by group and time. **(A)** Somatosensory function was improved from pre-to post-treatment (*P_corrected_* = 0.043) across groups. **(B)** RMT was negatively associated with somatosensory function at post-treatment across the two groups. ^*^P_corrected_ < 0.05.

In terms of the primary outcome, a two-way ANOVA indicated a significant main effect of time on somatosensory function (F_1,13_ = 5.05, *p* = 0.043, $\eta_{p}^{2}=0.28)$ (Figure 2A). Results indicated that both contralesional 1-Hz and ipsilesional 10-Hz stimulation improved somatosensory function from pre-to post-treatment (Mean_pre_ = 47.52, Mean_post_ = 50.17, *P_Bonferroni_* = 0.043). No other main or interaction effect was observed (all *P_Bonferroni_* > 0.05). Correlation analyses indicated that RMT was negatively associated with somatosensory function at post-treatment across the two groups (*r* = −0.63, *p* = 0.012, *n* = 15) (Figure 2B).

**Figure 3:**
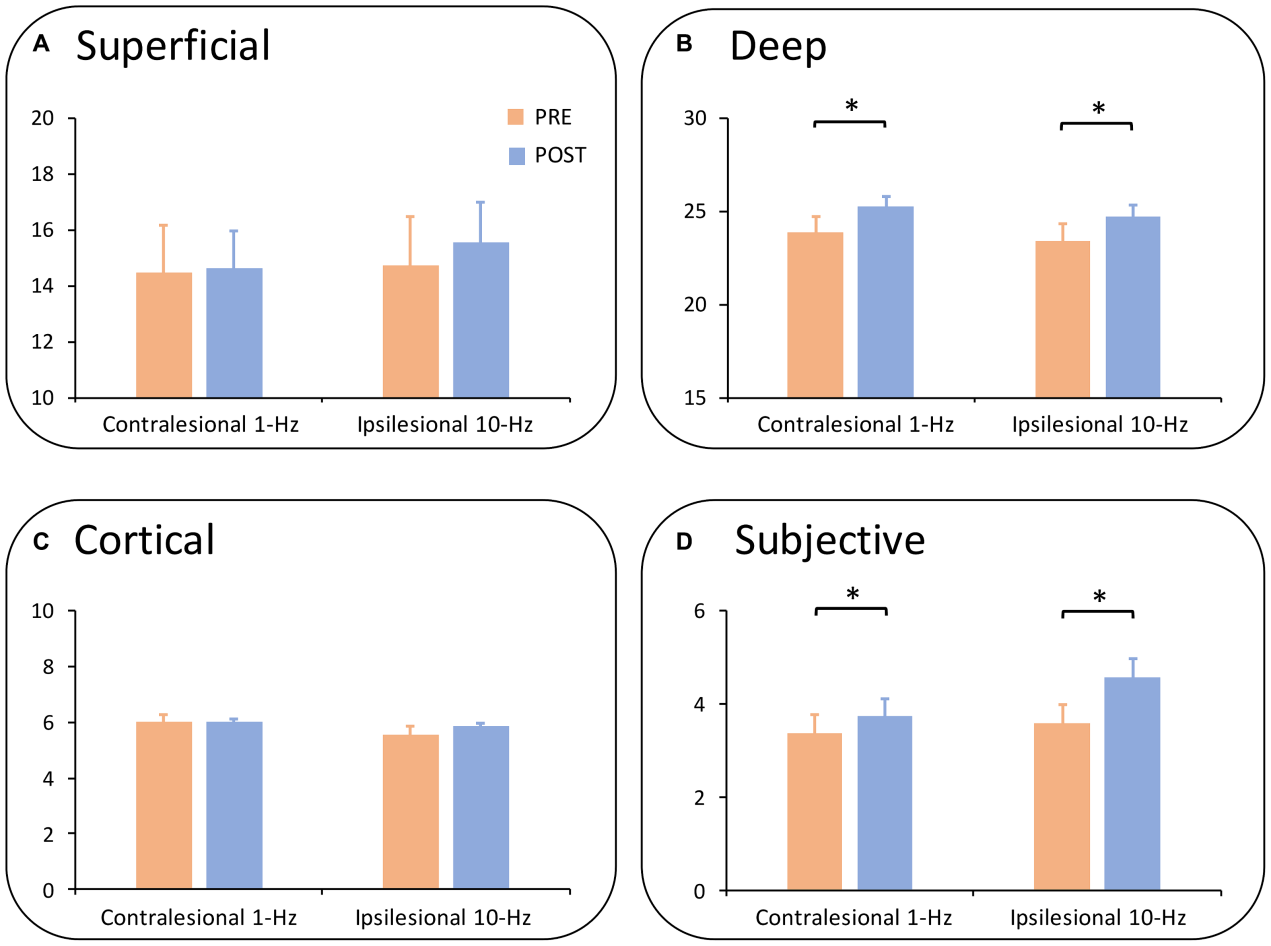
Subdimensions of somatosensory function by group and time. **(A)** Shows the results of superficial sensations. No significant main or interaction effect was observed. **(B)** Demonstrates the effect of deep sensations. Deep sensations were improved from pre-to post-treatment (*P_corrected_* = 0.020) across groups. **(C)** Illustrates the results of cortical sensations in which no significance was found. **(D)** Shows subjective sensations whereby both groups increased subjective sensations from pre-to post-treatment (*P_corrected_* = 0.009). ^*^P_corrected_ < 0.05; ^**^P_corrected_ < 0.01.

When the somatosensory function was split into subscales, a two-way ANOVA revealed no significant main or interaction effect in superficial sensations or cortical sensations (all *P_Bonferroni_* > 0.05) (Figures 3A,C). Meanwhile, there was a main effect of time on both deep sensations (F_1,13_ = 6.97, *p* = 0.020, $\eta_{p}^{2}=0.35)$ (Figure 3B) and subjective sensations (F_1,13_ = 9.29, *p* = 0.009, $\eta_{p}^{2}=0.42)$ (Figure 3D). Results indicated that both treatments improved deep sensations (Mean_pre_ = 23.65, Mean_post_ = 24.98, *P_Bonferroni_* = 0.020) and subjective sensations from pre-to post-treatment (Mean_pre_ = 3.47, Mean_post_ = 4.16, *P_Bonferroni_* = 0.009).

### 3.3 Corticospinal excitability

**Figure 4:**
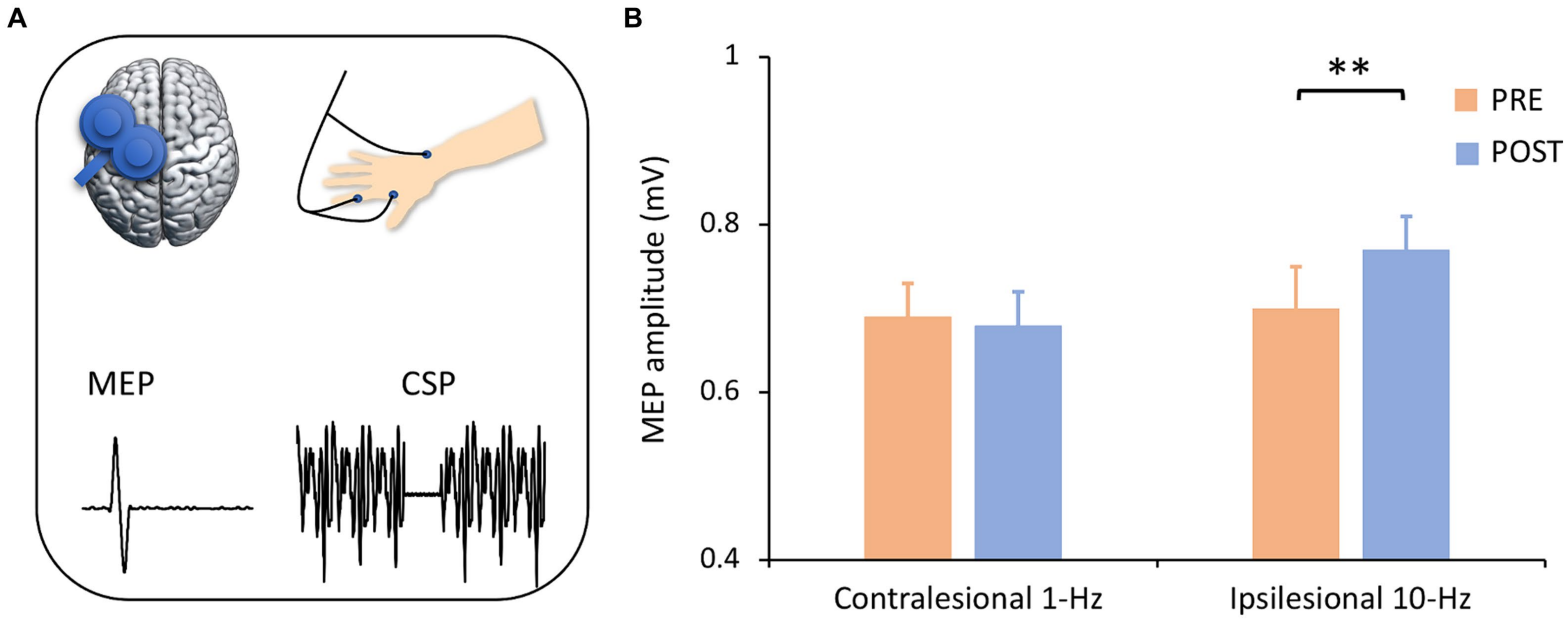
Corticospinal excitability by group and time. **(A)** Corticospinal excitability was assessed with single-pulse TMS-evoked potentials. It is noted that only left motor cortex was presented for illustrating purpose. **(B)** ANOVA analysis revealed an interaction effect, in which ipsilesional 10-Hz rTMS increased MEP amplitude from pre-to post-treatment (*P_corrected_* = 0.004) but contralesional 1-Hz stimulation did not. ^**^P_corrected_ < 0.01.

MEP data indicated a significant interaction (F_1,13_ = 11.94, *p* = 0.004, $\eta_{p}^{2}=0.48)$ (Figures 4A,B). *Post-hoc* comparisons indicated that ipsilesional 10-Hz stimulation increased MEP amplitude from pre-to post-treatment (Mean_pre_ = 0.70, Mean_post_ = 0.73, *P_Bonferroni_* = 0.004) while contralesional 1-Hz did not (*P_Bonferroni_* > 0.05). In terms of CSP latency, no significant main (F_1,13_ = 0.01, *p* = 0.971) or interaction effect (F_1,13_ = 0.21, *p* = 0.658) was observed.

### 3.4 Safety assessment

There was no serious adverse effect by monitoring patients’ vitality, physical and mental health. There was a slight chance to experience mild headache (0 and 0 in the 1-Hz and 10-Hz group) and/or mild scalp discomfort (0 and 1 in the 1-Hz and 10-Hz group), but these sensations dissolved within minutes or hours. Overall, the treatments were safe and well-tolerated.

## 4 Discussion

This pilot trial investigated two forms of S1-rTMS on somatosensory functioning in the acute and subacute phases of a stroke. Results indicate that both contralesional inhibitory and ipsilesional excitatory stimulation improved somatosensory functioning after a course of 10 sessions of treatment. Moreover, this effect was most prominent in deep sensations and subjective sensations. Using single-pulse EMG recordings, our data also revealed an increased MEP amplitude in the ipsilesional spinocortical pathway following ipsilesional excitatory treatment.

This pilot trial provided novel findings that S1-rTMS is able to improve somatosensory functioning in the acute and subacute phases of stroke. A few previous trials have demonstrated the benefits of S1-rTMS in the combination of peripheral therapies, although a sham peripheral condition was carefully designed. For instance, ipsilesional excitatory S1-rTMS combined with sensory stimulation or feedback improved sensory recovery following stroke compared to control conditions (Brodie et al., 2014; de Freitas Zanona et al., 2022). Our data confirmed the treatment effects on somatosensory functioning in the absence of peripheral therapies, thus providing further evidence to support the effects of S1-rTMS.

It is worth noting that lesions were mainly located in the subcortical regions in our patients, with three patients presenting temporal or frontoparietal lesions. Supplementary analyses were thus performed, which confirmed that improvement in somatosensory functioning was consistent when the three patients were removed from the analysis (Mean_pre_ = 48.17, Mean_post_ = 50.58, *P_Bonferroni_* = 0.050). Although there was only a trend effect due to decreased sample size, the effect size was getting larger ( $\eta_{p}^{2}=0.32\;vs.\;0.28$ ).

More importantly, we provided the first line of evidence that contralesional inhibitory rTMS is also effective in improving post-stroke somatosensory functioning (Figure 2). In motor recovery, contralesional inhibitory M1-rTMS has a definite effect while ipsilesional excitatory stimulation has a probable effect (Lefaucheur et al., 2020). In terms of S1-rTMS, two available studies have consistently delivered ipsilesional excitatory stimulation (Brodie et al., 2014; de Freitas Zanona et al., 2022), whereby contralesional inhibitory stimulation on sensory recovery has not been reported. It is noted that contralesional inhibitory rTMS was demonstrated to enhance motor learning post-stroke (Meehan et al., 2011). Our findings demonstrate an equal efficacy between ipsilesional and contralesional S1-rTMS. These findings provide an alternative rTMS paradigm in managing post-stroke somatosensory deficits.

More specifically, both rTMS treatments have a more prominent effect on deep and subjective sensations than on superficial and cortical sensations. It is noted that different somatosensations had distinct ascending pathways (Schünke et al., 2006), and it is possible that the S1-rTMS here selectively interacted with some of these pathways. For instance, S1-rTMS may preferentially modulate the sensory pathways underlying motion perception and vibration. Nonetheless, these findings open new discussions on the effects of rTMS on distinct somatosensory modules. Moreover, they have specified the implications for the management of certain somatosensations following stroke.

Our data also provide insights into the mechanisms of S1-rTMS effects on sensory recovery. Our data indicated that ipsilesional excitatory stimulation increased the MEP amplitude of the ipsilesional motor cortex. In one way, ipsilesional excitatory stimulation may directly increase S1 excitability and thus improve somatosensation. There are dense anatomo-functional connections between the S1 and M1 (White and DeAmicis, 1977; Donoghue and Parham, 1983; Veinante and Deschênes, 2003). Our recent study identified an association between somatosensory functioning and corticospinal excitability (Gao et al., 2023). In another way, excitatory ipsilesional S1-rTMS may increase the excitability of the injured motor cortex and thus facilitate sensorimotor recovery (Kim et al., 2006). However, it is noted that contralesional inhibitory stimulation did not induce significant excitability changes in the ipsilesional motor cortex. One would assume a significant excitability change in the contralesional motor cortex or a rebalance between the ipsilesional and contralesional motor cortex. It is acknowledged that MEP was not measured from the contralesional motor cortex in this study. Future studies would be able to validate this argument with ECG recordings from the bilateral motor cortices. It is interesting to find that a lower baseline RMT was associated with better somatosensory functioning after treatment (Figure 2B). This finding highlights the potential of corticospinal excitability in the predicting of rTMS treatment effect.

There were some limitations in this study. The sample was small, and the results here are preliminary that require validation in larger studies. There was a lack of long-term follow-up in this study due to the nature of a pilot trial. Potential long-lasting effects need to be evaluated in future studies. It is acknowledged that a sham group was not designed in this study. Findings reported here are therefore not sham-controlled. This is considered as a preliminary and open-label trial, in which a sham group is needed in future studies to exclude potential placebo effects. In addition, a neuronavigation system was not available in this study. Although the S1 can be reliably targeted by this methodology, a neuronavigation system would be able to increase targeting accuracy and assist in the identification of disease-relevant brain connections and networks mediating positive treatment outcomes (Cash et al., 2021a; Cash et al., 2021b). Motor functioning was not systematically examined with Fugl-Meyer Assessment (FMA) here (Fugl-Meyer et al., 1975). Our patients generally had no complains about motor functioning and had normal or approximately normal muscle strength as indexed by the muscle strength.

Although our findings are preliminary, they provide insights for future studies. Large, sham-controlled studies with long-term follow-up are suggested to validate the effects of S1-rTMS on somatosensation following stroke. Our findings also direct to the interactions between the sensory and motor pathways in the effects of motor cortex stimulation on somatosensation (Borich et al., 2015; Bolognini et al., 2016; Gao et al., 2023). We used single-pulse EMG responses to measure corticospinal excitability changes. Our data presented interesting findings on the corticospinal pathway. Future studies with whole brain assessment of excitability changes would be able to reveal more neural pathways and their interactions associated with sensory recovery (Chung et al., 2017; Che et al., 2019; Ye et al., 2022).

## 5 Conclusion

In conclusion, this pilot trial study demonstrates the primary somatosensory cortex as a potential rTMS target in somatosensory recovery in the acute and subacute phases of a stroke. Moreover, the contralesional inhibitory and ipsilesional excitatory stimulation are equally effective in sensory recovery.

## Data Availability

The raw data supporting the conclusions of this article will be made available by the authors, without undue reservation.
